# Learning from Bacteriophages - Advantages and Limitations of Phage and Phage-Encoded Protein Applications

**DOI:** 10.2174/138920312804871193

**Published:** 2012-12

**Authors:** Zuzanna Drulis-Kawa, Grażyna Majkowska-Skrobek, Barbara Maciejewska, Anne-Sophie Delattre, Rob Lavigne

**Affiliations:** 1 Institute of Genetics and Microbiology, University of Wrocław, Przybyszewskiego 63/77, 51-148 Wrocław, Poland; 2 Laboratory of Gene Technology, Katholieke Universiteit Leuven, Kasteelpark Arenberg 21, box 2462, B-3001 Leuven, Belgium

**Keywords:** antibiotics, bacterial multidrug resistance, bacteriophage therapy, phage-encoded proteins application,

## Abstract

The emergence of bacteria resistance to most of the currently available antibiotics has become a critical therapeutic problem. The bacteria causing both hospital and community-acquired infections are most often multidrug resistant. In view of the alarming level of antibiotic resistance between bacterial species and difficulties with treatment, alternative or supportive antibacterial cure has to be developed. The presented review focuses on the major characteristics of bacteriophages and phage-encoded proteins affecting their usefulness as antimicrobial agents. We discuss several issues such as mode of action, pharmacodynamics, pharmacokinetics, resistance and manufacturing aspects of bacteriophages and phage-encoded proteins application.

## 1 INTRODUCTION

The idea of using bacteriophages to treat infections has been well known since bacterial viruses were discovered by Frederick Twort [1] and Felix d’Hérelle [2] at the beginning of the 20th century. A few years later, Alexander Fleming revealed an antibacterial activity of *Penicillium* *notatum* mould and the antibiotics era was began. A large-scale introduction and success of antibiotics resulted in decreased interest in phage research/applications as a potential antimicrobial tool for controlling bacterial infections. At that time, the broad application of antibiotics resulted in an extensive bacterial resistance to these drugs, which shows that alternative methods are needed for eradication of pathogens. A renaissance of research on the biology of lytic bacteriophages appears to be a promising approach as a treatment for bacterial infections, especially those caused by multidrug resistant. A phage cocktail has been commonly applied as alternative or as supportive treatment simultaneously with antibiotics, particularly in Eastern Europe. For many years experiments were conducted only on a small scale in several centers, including Wroclaw and Georgia [3-11]. The most detailed historical publications documenting phage therapy come from Polish work done in Hirszfeld Institute by Stefan Slopek's group [12-14]. Positive results were indicated by the eradication of *Escherichia, Pseudomonas, Proteus, Klebsiella *and* Staphylococcus* clinical strains of various infections among both humans and animal models [12, 13, 15-17]. Nowadays, phages have been proposed as natural antimicrobial agents to fight bacterial infections in humans, inanimals or in crops of agricultural importance. Moreover, phage encoded proteins such as endolysins, exopolysaccharidases, and holins proved their ability as a promising alternative antibacterial products [18-21]. In this review, we concentrate on both advantages and limitations of antibiotics, bacteriophages and phage proteins as useful tools for bacteria eradication. 

## 2 ANTIBIOTICS AND ANTIMICROBIAL DRUG RESISTANCE 

**Fig. (1):**
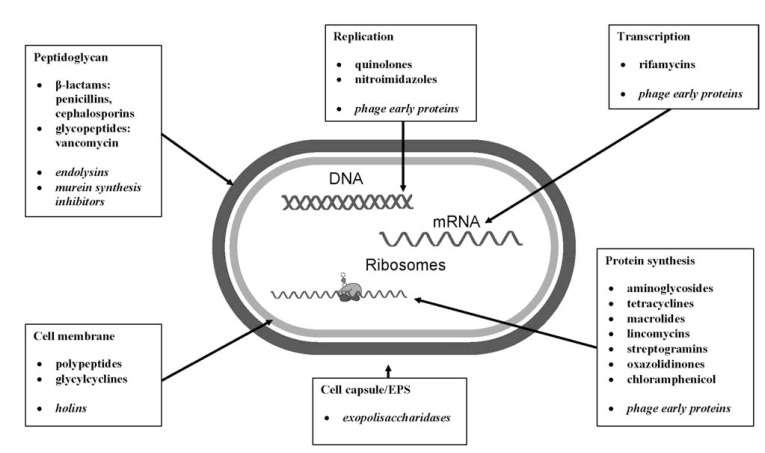
Mode of action of major antimicrobial agents and phage proteins (*in italics*).

Antibiotics are natural antimicrobial substances produced by certain groups of microorganisms, especially by bacteria: *Streptomyces*, *Bacillus*, and moulds: *Penicillium,* *Cephalosporium.* A prevalent number of therapeutic drugs are chemically modified derivatives of molecular versions produced by microbes (semi-synthetic antibiotics) designed to achieve better bactericidal properties. Currently, there are several groups of antibacterial drugs for clinical application including approximately 200 different compounds [22, 23]. Each of the chemical class exhibits a specific mode of action with a different activity spectrum (Fig. **1**). Some of antibiotics have a wide range of activity against aerobic and anaerobic Gram-positive and Gram-negative bacteria, including intracellular pathogens. A widespread use of antimicrobial drugs has led to the development of resistance in certain microorganisms. The most prevalent ones involve horizontally acquired genetic elements, thus susceptible population becomes resistant. The horizontal acquisition of resistance genes from another organism is implemented by: (i) transformation; (ii) transduction; and (iii) conjugation. The resistance elements encoded by chromosomal genes are transferred to plasmids by transposition, then forwarded to another related or not related bacterial species. Forwarded genes are multiplied or/and mutated which result in high expression of resistance. High resistance encoding genes may be transferred to multidrug cluster genes on R plasmids where 3,4,5 antibiotic groups resistome elements could be located. The use of antibiotics in medicine, veterinary, and agriculture contributes to the spread of R plasmids and multidrug resistant (MDR) strains dissemination [24]. Nowadays, severe clinical problems concern methicillin resistant *Staphylococcus aureus* (MRSA), *Enterobacteriaceae* (*Klebsiella pneumoniae, Escherichia coli, Enterobacter cloacae*) and pseudomonads (*Pseudomonas aeruginosa, Acinetobacter baumannii, Stenotrophomonas maltophilia*) producing ESBL (extended spectrum β-lactamases), MBL (metalo- β-lactamases) or KPC (*Klebsiella* carbapenemases) hydrolysis enzymes. There is a pandemic dissemination and clonal outbreaks of MDR strains. The greatest threat is the increasing evidence of R plasmid mediated resistance that have evolved efficient dissemination methods through the sensitive and susceptible bacterial isolates [25-29]. Treatment of Cystic Fibrosis (CF) linked pneumonia has been identified to induce temperate phages that carry antimicrobial resistance genes (resistome vehicles) and horizontally disseminate these genes through their specific bacterial host range in the lung environment [30, 31]. The R plasmids/temperate phages carrying resistomes as a principal cause of resistance transfer predate the antibiotic era especially in hospital environment where the high selective pressure of antibiotics is present. 

In the twilight of antibiotic usefulness several efforts can be made to reduce the emergence of antibiotic resistance: (i) control, reduce, or cycle antibiotic usage; (ii) improvement in hygiene in hospitals and among hospital personnel; (iii) discovery or development of new antibiotics; (iv) modification of existing antibiotics; (v) development of inhibitors of antibiotic modifying or hydrolysing enzymes; or (vi) development of alternative antibacterial therapies (for example phage therapy).

## 3 BACTERIOPHAGES 

Bacteriophages (phages) are viruses that infect bacterial cells. They can be found in every environment where their bacterial host are present. The population number of phages in aquatic systems was established in the range of 10^4^ - 10^8 ^virions per ml and in the soil and sediment particles of approximately of 10^9^ virions per 1g [32]. 

At present, over 5500 different bacteriophages have been discovered, each of which being able to infect one or several types of bacteria [33]. Generally, phages as obligatory parasites of a bacterial cell show several life cycles: lytic, lysogenic, pseudolysogenic and chronic infections [32]. The main interest in phage application as antimicrobials has focused on lytic tailed phages representing three families of *Caudovirales *order: (i) *Myoviridae* with the biggest capsid head (~150 nm) and contractile tail; (ii) *Siphoviridae *with a relatively small capsid head (~50-60 nm) and a long flexible, noncontractile tail; and (iii) *Podoviridae *with a small capsid head (~50-60 nm) and a short tail [34-36]. There are also some reports of some cubic phages (phiX174 and Qb) [37-39] or filamentous phages (M13 and Pf3) application [40, 41]. 

The mode of action of lytic phages as bacterial predators (lytic cycle) covers several crucial steps determining a specific ability to kill bacteria [34, 42-47]:

1. *Adsorption to specific receptor*. Phage attachment to a host cell is a highly specific process involving complementary receptors on the surface of a susceptible host cell and an infecting virus. There are two major types of receptors distinguished: surface components of a bacterial cell including lipopolysaccharide (LPS), peptidoglycan, teichoic acids, outer membrane proteins, oligosaccharides, capsule, IV type fimbriae, flagellum (for somatic phages) and sex pilus (for male-specific F^+^ phages). Some of the phages developed specific enzymes degrading exopolysaccharide (EPS) structures (capsule, slime), masking or covering a targeted receptor.
2. *DNA injection*. The DNA of lytic phages is transferred into the cell after peptidoglycan degradation (phage lysozyme activity) and pore formation in the bacterial cell wall.
3. *Redirection of host metabolism to phage DNA replication and phage protein synthesis*. After DNA penetration of the cytoplasm, the expression of phage early genes redirects the host synthesis machinery to the reproduction of viral nucleic acid and proteins.
4. Assembly and packing of phage particles.
5. *Bacterial cell lysis and phage progeny release*. Phage late proteins including lysins, holins or murein synthesis inhibitors are responsible for host cell lysis and virion burst to the environment.

Bacteriophages are highly specific, with most infecting only a single species of bacteria. To enter a bacterial cell, bacteriophages attach to specific receptors on the surface of the host cell. That specificity of interaction between phage attachment structures and host cell surface receptors mostly influences the bacterial host range [9, 32]. The specificity of predator-prey interaction limits the application of certain phages in the therapy, however, it ensures no influence on normal flora, because phages eradicate the targeted strain only, protecting bacterial colonizers unrelated to pathogen species. A broad antimicrobial spectrum of most drugs has an effect on a wide range eradication of infecting pathogens, but dysbiosis and secondary bacterial or fungi infections are possible.

The disadvantage of phage therapy in comparison to antibiotics is the need to determine an etiological factor causing an infection, by culturing a clinical sample first and then to identify a pathogen, using standard microbiology diagnostic procedures. This step is crucial in all cases to get the knowledge about targeted bacterial species. Even if we possess a collection of “ready to use” phage preparations we still need to know the “exact name” of bacteria for eradication. A microbiology diagnosis of clinical samples should also be common practice in antibiotic treatment, but because of probable limitations such as time (severity of infection), cost, or limited availability of a clinical laboratory in the vicinity of physician’s practice, antibiotics are prescribed on the basis of infection symptoms, current knowledge of pathogen prevalence in a particular type of infection and the occurrence of antibiotic drug resistance among certain species. The phage choice for application is usually based on: (i) selection of several most potent phages from an available collection (ready to use phage cocktails); (ii) assortment of effective phages from an available collection after phage typing of isolated bacteria (composed phage cocktails); or (iii) in extreme situations, when no active phage is present against a severe pathogen, the lytic phage may be found, isolated directly from environment and then prepared for application. What also has to be emphasized is an enormous number of phage variety as effective antimicrobials. The abundance of environmental phages gives us a boundless opportunity to isolate and compose phage preparation against all kinds of current and future bacterial pathogens regardless of resistance development [32, 48]. Although standardized methods to generate phage cocktails have been proposed [49], no clear official guidelines exist [50]. In phage choice and selection as potential safe antimicrobials, detailed comprehensive characteristics of genetic (genome sequencing) and phenotypic properties has to be performed, whereby unfavourable features such as lysogeny-associated genes, toxin or enzyme encoding genes presence could be detected [48]. Although the sequencing methods are now widely available and the phage genome data bases contain a big number of identified phage genes, there is still a lot of hypothetical, putative protein with a predicted or unknown function detected in most of viral genomes.

Antibacterial substances used in therapy should exhibit desirable properties named as efficient pharmacodynamics (PD) and pharmacokinetics (PK) [51]. The term of PD describes the ability of a drug to eliminate bacterial cells but also points at the influence on host tissues and organs. Antibacterial efficacy of phages depends on virulence ability consisting of duration of phage generation time including efficient adhesion, latent period and virion release. The number of phage progeny produced during one life cycle determine the rate of phage population growth. The shorter generation time, higher adsorption rate to certain bacteria and higher burst size, the better antibacterial efficacy. Phage virulence may vary under certain conditions: (i) multiplicity of infection (MOI), which determines phage titer-dependent killing; and (ii) current state of bacterial population, thus phages are most effective on host population in an exponential phase of bacterial growth in comparison to stationary or adaptation phases [48, 52, 53]. In comparison to phages drugs exhibit concentration-dependent killing (aminoglycosides, quinolones, daptomycin) or time-dependent killing (β-lactams, tetracyclines, glycylcyclines, macrolides, ketolides) and based on antibacterial efficacy we may distinguish bacteriostatic (inhibiting cell proliferation) or bactericidal (killing) antibiotics [54]. Lytic viruses propagated on the host lead to the lysis of the cell in the final step of phage life cycle. Rapid lysis of a big number of cells and release of LPS from Gram-negative bacteria in a short period of time may cause serious side effects on the host, however, similar effects may occur during bactericidal antibiotic utility, as well [55]. Similarly to antibiotics, the effective phage concentration at the site of infection and current state of bacterial population strongly affect therapy success. Phages are described as self-replicating antibacterial agents, because the phage titer increases at the infection site until the bacterial host is present and efficient concentration may be achieved at exact body/ tissue location where it is needed [56]. These features are invaluable in treatment of local infections such ulcerations, burn wounds, diabetes foot infections. The translocation of phages is relatively easy thus the concentration of viruses is maintained locally until the bacteria are present. In contrast to phages, effective concentration of antibiotics at the site of infection is difficult to obtain because of low blood circulation, and pus-antibiotic interaction decreasing its activity [56-58]. Another issue of PD is the efficacy of phage therapy in terms of pathogens localization. In contrast to antibiotics, no phage preparations for intracellular pathogens are available, however, there are reports on *Mycobacterium* eradication located inside macrophages by TM4 phage in combination with non pathogenic *M. smegmatis* co-infection, but the exact mechanism of this phenomenon has to be elucidated [59]. 

The second feature describing antimicrobial potency is PK [51]. The PK refers to what the treated organisms do with the drug. The PK concerns: (i) absorption; (ii) distribution (administration efficiency, penetration to particular tissues), (iii) metabolism (shelf-life, mechanisms of metabolic modification); and (iv) excretion (mechanism of elimination). Molecular weight, ionization, solubility, and formulation of antibiotic affect the absorption and bioavailability of a drug depending on a route of administration. Most antibiotics can be applied orally because of high bioavailability (intestine - blood system penetration). The concentration at the infection site is related to systemic concentration and blood circulation thus is influenced strongly by the body metabolism in lungs, blood, or liver. Bacteriophage penetration and distribution in animal body differs from antibiotic distribution. Phage size and virion composition also determine distribution and tissue penetration. There is an apparent discrepancy regarding phage absorption and distribution reported in relation to the route of administration. Some authors evidenced an effective phage translocation to the systemic circulation after local or oral application, but others restricted the local or *per os* administration only to topical and gastrointestinal infections, respectively [57, 60]. The phage PK was presented in details by Dabrowska and co-authors [60]. Bacteriophages are seen by the immune system as potential invaders (viruses) and they are rapidly eliminated from the systemic circulation by reticuloendothelial system (RES) clearance (innate immune mechanisms), then accumulated in spleen and liver or/and inactivated by adoptive immune defense mechanisms involving immunoglobulin in the case of repetitive application [60, 61]. Such rapid clearance affects self-limitation of phage titer in animal fluids and depends on the major capsid protein structure/composition, thus an effective phage concentration in systemic circulation can be achieved and maintained by modified long-circulating virions [62]. The majority of phage therapy research working on treatment efficacy focuses on achieving proper phage concentration (MOI aspects) and timing of administration, which are crucial elements to establish for each particular phage in used cocktail [48, 52]. The virion stability issue of certain phage representatives in terms of their susceptibility to different external physical and chemical factors, such as temperature, acidity, and ions was described recently by Jończyk [63], however, in other papers there is not much stability data of utilized phage preparations [52]. Available formulations of phage preparation are relatively limited in scientific literature, examples are known for liquid phage filtrate, tablets, and formulas for local application regarding a required route of application [52, 57].

### 3.1 Bacterial Anti-Phage Resistance Mechanisms 

Bacteria as phages prey have evolved several adaptive mechanisms protecting the cell from viral infection. Comprehensive reviews concerning bacteriophage resistance mechanism have been published in recent years [21, 64, 65]. Bacteria can inhibit the phage cycle on crucial steps of propagation process by: (i) preventing phage adsorption; (ii) preventing DNA integration by Superinfection exclusion system (Sie); (iii) degradation of phage DNA by Restriction-Modification (RM) defense system and Clustered Regularly Interspaced Short Palindromic Repeats (CRISPR); and (iv) blocking phage replication, transcription, translation or virions assembly by Abortive Infection system (Abi). The most common resistance mechanism to phage infection is due to the lack of a bacterial receptor, which blocks phage adsorption on the bacterial surface and results in complete loss of ability to generate virus progeny. The lack of the receptors can be caused by structural modification or masking of the target as was noticed for *E.coli* outer-membrane protein TraT, modifying the conformation of outer-membrane protein A (OmpA), a receptor for T-even-like phages [66] and for *Staphylococcus aureus* protein A covering the phage receptors [67]. The loss of receptors can also occur through phase variation of the host where the cell surface composition is changed. Such phenomena was desribed for *Bordetella spp*. Bvg+ versus Bvg- phase [68] and *Shigella flexneri* 1b serovar versus 3b serovar [69]. In an adaptive response the adhesion inhibition can be implemented by EPS production found in environmental *Pseudomonas spp., *rods (alginates) or glycoconjugates synthesis by *Enterobacteriaceae* (capsules), even as temporally phenomenon [70]. Connerton and co-authors reported the impact of phage predation on genomic rearrangement of *Campylobacter jejuni* to insusceptible population [71]. The bacteriophages as a permanent partner of bacteria have evolved and adapted to extracellular matrix as to new receptors or by production of EPS degrading enzymes (discussed below). 

The anti-phage bacterial resistance is accomplished by mutation and selection or temperate phage acquisition and spread vertically from parental to daughter cells. The horizontal dissemination is also possible by induction and propagation of temperate phage or, in the case of resistance genes located on moving element such as plasmid, by conjugation process [72]. The prevalence and probability of development in phage resistance is ambiguous because many publications pointed out a low level of induced resistance during phage therapy (resistance frequency of 10^-8^) [6, 8, 57] in contrast to some *in vitro* analysis (10^-4^ - 10^-8^). The discrepancy between clinical observations and *in vitro* analysis indicate that the impact of immune system activity, environmental condition variation and the virulence of particular phage have to be taken into account [73-75]. 

Unlike antibiotics, the remediation of the antiphage resistance problem is relatively fast and easy. Isolation of novel active phages from environmental sources or progressive adaptation of viral parasite to resistant host population are possible. Phages evolve simultaneously with bacteria following changes among resistant clones [32, 55]. However, in common phage therapies, cocktails of phages (3-5) are applied to prevent possible resistance development. Another reason for using a cocktail of phages is that they probably all infect through different cell receptors increase targeting of the cocktail and alteration in surface display of epitopes associated with phage adsorption. Similarly to antibiotics, the composition of several phages against one type of bacterial pathogen in the treatment affects an increase in activity spectrum and may have synergistic effect [55, 57]. 

## 4 PHAGE-ENCODED PROTEINS 

The major mechanisms of phage infection may be used as a model for antimicrobials design. Bacteriophages are able to produce special proteins involved in the crucial steps of lytic cycle strategy. Particular phage proteins may destruct bacterial envelopes such as: peptydoglycan, cell membrane, and cell capsule, key elements protecting the bacterial cell from viral infection and progeny release. There are also phage-encoded proteins affecting bacterial DNA replication, transcription, protein synthesis and cell division by the protein-protein interactions (Fig. **1**). 

### 4.1 EPS- Degrading Phage Enzymes

To entry their genome into the host cell where their genetic information is expressed and replicated, bacteriophages infecting polysaccharide slime or capsule surrounded bacteria, have evolved the ability to overcome the EPS structure by producing virion-associated proteins with polysaccharide depolymerization activities. The EPS depolymerases of the phages infecting, for instance, encapsulated *E. coli* K1 [76] and K95 strains [77], *V. cholerae* O139 strain [78], *P. aeruginosa* strain [79], *P. agglomerans* strains [80] and *P. putida* strain [81] specifically recognize the bacterial polysaccharides as primary receptors involved in adsorption phage processes to the host cell and subsequently degrade them what is necessary to complete the lytic cycle. Utilising polysaccharide depolymerases and depolymerases of lytic phages is a promising yet challenging antimicrobial therapy. The main area that shows the greatest potential of this application is to prevent and control of biofilm-associated clinical and industrial aspects [82]. The EPS depolymerases target the EPS matrix of biofilm by destroying its physical integrity allowing the phage to gain access to bacterial cells hidden inside. Moreover, dispersing EPS from the biofilms can also result in the release of bacterial cells, making them more accessible to the effect of antibiotics or natural defense mechanisms [83].

Depending on catalytic activity, phage-borne depolymerases may be classified into two groups: (i) hydrolases (also known as polysaccharases); and (ii) polysaccharide lyases. The first of these enzymes act hydrolytically, breaking the glycosyl-oxygen bound in the glycosidic linkage. Lyases, in turn, cleave the linkage between monosaccharide and the C4 of uronic acid with a simultaneous introduction of an unsaturated bond between the C4 and C5 of the non-reducing uronic acid terminal [84]. Some recent examples of these enzymes have been reported in Table **1**. Depolymerases can occur in two forms: one as a freely diffusible protein, and second as a component of phage particles, mainly as tail spike or tail fiber proteins [85, 86]. 

The most widely studied type of the EPS depolymerases is endosialidase or endo-N-acetylneuraminidase (endoN), (EC 3.2.1.129, a glycosyl hydrolase) of bacteriophages specific for human pathogenic *E. coli* K1, which specifically recognizes and hydrolyzes internal α-2,8-linkages in polysialic acid (polySia), a polymer which forms a capsule of the host. So far, more than 20 K1-specific phages that contain enzymes with capsule-depolymerizing activities have been reported in the literature [87-94]. Deszo and co-workers [94] first described *E. coli* K1 prophage CUS-3, a temperate bacteriophage with an endosialidase gene. The genes encoding enzymatic active endosialidases have been cloned from several, phages (K1A, K1E, K1F, 63D, K1-5, CUS-3 (Table **1**), [93, 95-101]. Interestingly, endosialidases were found only as tail spike proteins in phages specific to *E. coli* and other similar polysialic-acid degrading enzymes have not been discovered in any other organism [101]. Intensive studies have also focused on the EPS depolymerases associated with both pseudomonad [79, 81, 102-105] and *Klebsiella* phages [106-111] that are able to degrade an alginate surrounding cell and a thick polysaccharide capsule, respectively. Hyaluronate lyases constitute another type of phage-associated enzymes, essential for penetration of host mucoid capsule. This class of endoglycosaminidase enzymes (EC 4.2.2.1) is capable of degrading hyaluronate, a sole component of the capsular material of *Streptococcus pyogenes* and *Streptococcus equi*, into unsaturated disaccharide units [112-117]. In contrary to the mammalian hyaluronidases which promote the growth of carcinoma cells, the phage hyaluronidase with unique enzymatic activity can be exploited in suppressing hyaluronan-mediated tumor growth and progression [118]. Most of the genes encodes hyaluronidases are located in the prophages, which are inserted into the genomes of the bacterial strains. 

Although the capacity of phages to produce polysaccharide depolymerase enzymes was first reported in 1929 [119], it was not until 2002 that Sutherland and co-workers suggested that phage and phage-derived depolymerases should be used as therapeutic agents to eradicate bacterial biofilms. In one of their preliminary reports, a complete eradication of *Enterobacter cloacae* biofilms by a cocktail of three phages capable of degrading the extracellular polysaccharides within the biofilm and killing the bacterial cells was described [120]. The same authors have confirmed the key role of the phage-borne depolymerases in disrupting the EPS of the phage-susceptible mono-species biofilm in another study [121]. In turn, Hanlon *et al.* [122] have proved that phage diffusion through *P. aeruginosa* biofilm may be facilitated by a reduction in alginate viscosity brought about by enzymatic degradation. 

Most phage depolymerases act only against the bacterial species from which the phage was originally isolated and rarely degrade more than a few closely polysaccharide structures [84, 123]. An interesting example of a phage that has a dual host specificity based on encoding two different enzymatic tail fiber proteins is phage φK1-5. The first protein is an endosialidase, which allows this phage to attach to and degrade the K1 polysaccharide capsules, whereas the second is a lyase. K5 lyase specifically cleaves the *E. coli* K5-capsular polymer composed of the disaccharide repeating unit of N-acetyl-heparosan-4-GlcA-(β1,4)-GlcNAc-(α1), [93]. Another example is coliphage φK5-95, which also encodes K5 lyase A (KflA), [124]. Interestingly, Kf1A is the first described viral tail spike protein containing a single-stranded β-helix domain with a lyase catalytic site which shows similarity to bacterial glycosaminoglycan-degrading enzymes. The specificity of biofilm-degrading enzymes for host bacteria is both an advantage and a disadvantage for therapy. Owing to their high specificity to the bacterial EPS, these enzymes can be applied to achieve the disruption of the existing bacterial biofilm, with minimal destruction of the beneficial microbial flora as well as human cells, which gives them the supremacy over antibiotics [73]. On the other hand, the host specificity of phages associated with depolymerases as therapeutic agents against biofilm requires: (i) the isolation of the biofilm organisms and screening against a bank of phages; (ii) the creation of a bank of well-characterized phages; (iii) the existence of appropriate production, purification and application protocols [125]. Nevertheless there has to be a starting point, but biofilms in the chronic lung diseases associated with CF, Chronic Obstructive Pulmonary Disease (COPD) and non-CF bronchiectasis are mixed microbial communities and thus targeting of a subsection of the bacterial community may be enough to destabilise biofilm formation and subsequently limit the clinical exacerbation state [126].

Lu and Collins [127, 128], using novel synthetic biology technologies, presented a methodology to engineer enzymatic active phages that are both capable of killing the bacteria in species-specific manner by lysis and dispersing the EPS matrix because they have been also engineered to express the most effective EPS-degrading enzymes. It was demonstrated that the engineered *E. coli* T7 phage to express dispersin B (DspB), an enzyme that hydrolyzes a crucial biofilm EPS component, β-1,6-Nacetyl-D-glucosamine [129], is 100-fold more efficient at removing bacterial biofilms than non-enzymatic T7 phage alone. What is important, the enzyme was released into the extracellular milieu following the bacterial cell lysis, and therefore exogenous administration of the enzyme, which could cause side effects is not required [127]. In some cases, phages can induce the expression of enzymes that degrade the biofilm matrix by the bacterial host [44]. However, on the other hand, the enzymatic treatment of biofilm, particularly multi-species biofilms can also strengthen the EPS structure [130].

Using modern technology could potentially broaden the phage host range to target a wide range of biofilms. For instance, a modified T7 phage to express two enzymatic proteins, lyase and endosialidase, attached to its surface could be replicated in both *E. coli* B and *E coli* that produce both K1 and K5 polysaccharide capsules [128]. Furthermore, utilizing other enzymes such as DspB, focused on adhesins present in different bacterial species, including clinical strains which are crucial for biofilm formation, phages could be constructed with a much wider range of biofilm activities. Based on this modular design strategy, these researchers proposed creating a diverse library of biofilm-dispersing phages that would be more useful in therapy than isolation of natural phages with polysaccharide depolymerization activities from the environment. Moreover, these engineered enzymatic active phages may also be applied as phage cocktails as well as in combination with one or more conventional antimicrobial agents against bacterial biofilms in medical, industrial and biotechnological settings [128, 131,]. Lu and Collins [132] demonstrated engineered temperate bacteriophage with overexpression of proteins that enhance the effectiveness of antibiotics. It was shown that such phage may augment the killing of multidrug-resistant bacterial strains, bacteria embedded within a biofilm matrix and act as a strong adjuvant for other antibiotics (e.g., aminoglycosides and β-lactams). A promising results regarding combination of the enzymatic-active native bacteriophages with other anti-biofilm treatments was already proposed [133] and it turned out that the application of *Klebsiella* phages with the antibiotics displayed a synergistic effect [109, 110]. In these studies, a significant reduction in bacteria numbers of both younger and older *K. pneumoniae* biofilms was observed after treatment with depolymerase producing lytic bacteriophage (KPO1K2) and ciprofloxacin.

Apart from preventing and eradicating biofilm formation, phage-borne depolymerases have also been used for other various purposes. It was shown that these purified enzymes can be potential tools to reduce and even eliminate colonization of fire blight host plants by *Erwinia amylovora* [134], to treat systemic infections caused by encapsulated bacteria in animals [135, 136] as well as to increase susceptibility of *Bacillus anthracis* to phagocyting killing, and to decrease its virulence [137, 138]. They can also provide a promising alternative to antiserum for typing bacterial strains [139]. As phage-borne endosialidase are currently the only known enzymes that specifically degrade polySia, they are very useful in polySia research, including neurobiology and oncobiology [140-142]. Moreover, these enzymes can be applied to induce degradation of artificial polySia-based hydrogel in well-defined time points, which is used as a scaffold material for tissue engineering [143]. More information regarding the significance of endosialidases in medical and biochemical applications can be found in the recent review published by Jakobsson and co-workers [144].

### 4.2 Phage Early Proteins

About 64% of the protein-protein interactions between bacteriophages and their hosts occur in the early stages of infection, i.e. during the transition from a host to a phage-oriented metabolism [145], which makes the so-called early phage proteins of primary importance in the infection cycle. The vast majority of these proteins are smaller than 250 amino acids, and their expression seems to be triggered by specific environmental conditions [146]. Some early genes are indeed very lethal to the host, which is why deciphering the role and the interaction mechanisms of these genes (and the proteins they encode) is a powerful way to discover new antibacterial strategies. The consequences of the protein-protein interactions between phages and bacteria are: inhibition, stimulation or redirection of host proteins to a step in the phage infection cycle. The phage early proteins target several host machineries, including DNA replication, transcription, protein synthesis and cell division (Table **2**), as shown here by some recent examples: 

#### 4.2.1 Influence on Bacterial Replication

Increasing evidence indicates that host DNA replication is targeted by phages. *Staphylococcus aureus*-infecting phage proteins were shown to directly interact with the host replication apparatus by protein-protein interactions, targeting DnaG (involved in replication initiation), PT-R14, DnaI (the helicase loader) and DnaN (the sliding clamp) [147]. Most lytic phages encode their own genome replication machinery. For example, phage T4 encodes 10 proteins that will form the phage replisome [148]. On the contrary, temperate phages need the host DNA replication machinery [149]. Nevertheless in both cases phages either sabotage host replication to shut it off, or redirect it to replicate their own genome. For example, lytic phages shut off the host replication by direct interactions between early phage proteins and host proteins involved in DNA metabolism. In addition, phages also degrade the host nucleoid to provide precursors for the phage genome replication. The temperate λ phage protein P interacts with the DnaB protein of *E. coli* [150], thus inhibiting its ATPase activity and its interaction with the host DNA primase, eventually leading to blocking of replication. From an antibacterial point of view, extensive work on this phenomenon is of primary interest in the search for novel DNA replication hijacking mechanisms. Moreover, the targeting of host DNA replication by phages yields a huge diversity of inhibition mechanisms, and as a consequence a huge diversity of susceptible drug targets. 

#### 4.2.2 Influence on Bacterial Transcription

Bacteriophages aim at favoring their own transcription while reducing that of the host. As a consequence, most phages use or interact with the host transcription apparatus during infection to shut down, inhibit or redirect. Through evolution, several strategies have been evolved by phages to impact the host bacterial RNA polymerase complex (RNAP). Some of them encode their own σ factor, which will replace that of the host in the RNAP complex. The phage early genes are thus transcribed by the bacterial RNAP complex containing the phage σ factor (late phage genes bare bacterial-like promoter sequences). In the case of the well-known *E. coli*-infecting phage T4, the host RNAP complex is recruited and used for phage genes transcription. Bacteriophages can also shut off the host transcription, as in the case of T7: the host RNAP is inhibited and phage genes are transcribed by the phage-encoded RNAP [151, 152]. Recently, a novel *E. coli* phage was identified [153]. The φEco32 phage was isolated in Tbilisi, Georgia, and belongs to the *Podoviridae* family. It infects *E. coli* 55, a bacterium isolated from a cow suffering from mastitis. Interestingly, φEco32 lyses more than 95% of *E. coli* strains isolated from mastitis-suffering cows and is thus of major interest to design a new phage-based antibacterial strategy. The main characteristic of this phage is the presence of two proteins involved in host RNAP hijacking (gp36 and gp79). The phage genome encodes two types of promoters that are recognized by the host RNAP complex containing either the α70 factor (early genes), or the phage protein gp36 (for middle/late genes). The gp36 protein is indeed a putative σ factor. The gp79 protein inhibits transcription by the host RNAP complex *in vitro*. Even though the φEco32 phage does not infect humans, deciphering the protein-protein interactions between host and phage can lead to characterization of transcription inhibition and thus improve the design of phage-based antibacterial compounds.

#### 4.2.3 Influence on Bacterial Protein Synthesis

Because they are present in high quantity in the bacterial cell, phages use the host ribosomes to translate their own mRNA into proteins [154]. But phages also do influence the host translation machinery. For example, the φYS40 phage infecting *Thermus thermophilus* produces most of its middle/late mRNA transcripts without leader sequence (which usually contains the ribosome binding sequence). Nevertheless, this phage is able to direct the host translation apparatus to translate mRNA without leader sequence. Several lines of evidence indicate that to do so, the phage upregulates expression of IF2 while downregulating expression of IF3, thus stimulating translation of leaderless mRNA [155, 156]. Another example is the well-studied *E. coli*-infecting T4 phage which encodes ADP-ribosyltransferases, like ModB, which ribosylates host proteins involved in translation [157, 158]. No evidence is available yet on their influence on translation. In the case of the T7 phage, the gp0.7 protein phosphorylates seven proteins involved in translation, which could influence host translation, and this leads to the perspective of characterizing new phage interference with mRNA translation. However, detailed interaction analyses are still lacking. From an antibacterial point of view, it is indeed difficult to imagine a way to mimic these inhibitory interactions, but deciphering these mechanisms offers a new view on interfering with essential cellular mechanisms in bacteria. 

#### 4.2.4 Influence on Bacterial Division

The bacterial cell division is obviously an essential step during the cell growth. This step is indeed the target of several antibiotics. It was shown that some phage-encoded proteins also inhibit this process [152]. For example, the prophage Rac uses the Kil protein to seriously impair *E. coli* cell division by interacting directly or indirectly with FtsZ. Moreover, homologs of the Kil protein were found in other phages [159-163]. Nevertheless, evidence of a direct effect of these interactions on bacterial cell division is still lacking. 

#### 4.2.5 Phage early proteins and antibacterial therapy

The intricacy of the examples listed above indicates that target-molecule selection can be complemented using the knowledge we have gained from the study of bacteriophages by exploiting their unique strategies to arrest critical cellular host processes. 

Extensive work on the interactions between phage and host proteins can lead to development of new growth-inhibition strategies. An innovative high-throughput screening strategy based on *S. aureus*-infecting phages was designed by Liu and co-workers [147]. *S. aureus* is indeed a harmful human pathogen and a major public health problem being responsible for numerous nosocomial infections. Moreover, the emergence of methicillin-resistant clinical isolates (MRSA) prompted the development of new antibacterial strategies. Liu and colleagues focused on phage proteins causing bacterial growth inhibition. After identification of the host interaction partner, they aim at using chemical compounds that would mimic these phage-host interactions as new antibacterials. In other words, the phage proteins were used to validate putative targets in the host cell and to screen for efficient inhibitors. The genome of the 27 *S. aureus*-infecting phages were sequenced and annotated, 964 ORFs were cloned in an inducible expression vector, and proteins having a toxic/inhibitory effect on host cell growth were selected further. To find the interacting partner of these proteins and to confirm the interaction, several *in vivo* and *in vitro* protein-protein interaction assays were performed (Yeast Two Hybrid, Far Western Blot, Surface Plasmon Resonance, TR-FRET). 31 polypeptide families were identified as host growth-inhibiting. They mainly influence the host replication and transcription apparatus, as exemplified by the interaction between ORF104 of phage 77 and the DnaI host protein (the helicase loader), which is essential for DNA replication initiation. 125,000 chemical compounds from commercially available libraries were screened, and among them 36 were found to be inhibitors of the ORF104 phage 77-DnaI interaction with an IC50 lower than 10µM. None of them is cytotoxic to human primary hepatocytes. Liu and colleagues indeed showed the effectiveness of this high-throughput platform for phage-based antibacterial compounds. This approach can be used as a screening tool for other bacteriophages and other bacterial pathogens.

However, implementations by industry have remained limited, despite the body of available research and the vast untapped potential. That said, the bacteriophage research field remains strongly genome-oriented and is often limited to modeling organism hosts. In addition, the step towards product development is particularly long, in view of fundamental research which precedes the selection of a suitable bacterial target candidate and relevant phage-based inhibitors. This research is also dependent on technically challenging approaches including crystallization of target and phage proteins and rational small molecule design. Nevertheless, we can project an increased need for sustainable new antibacterials in the future, which could tilt the balance towards supporting fundamental exploratory research in this field, focusing on proteome and metabolomics based research. 

### 4.3 Endolysins - Phage Enzymes Degrading Peptidoglycan

One of the most popular strategies for bacterial cell lysins among Gram-positive specific phages is utilization of two proteins combination: endolysin degrading peptidoglycan and holin disrupting cell membrane [164]. These two proteins are also used by some well-studied Gram-negative phages T4, P1, T1, Mu and SP6 [165]. Many other Gram-negative specific phages like T7, P2, φKMV or λ phage besides of endolysins and holins encode other auxiliary co-enzymes: Rz and Rz1 which form the so-called “lysis cassette” [164-166]. Some of the phages are effective only with endolysins possessing extra cell binding domain or signal-arrest-release (SAR) domain [167-169] or both of them (fOg44 phage endolysin) [169]. The occurrence of endolysins operating with extra domain and no auxiliary proteins, such as *Listeria monocytogenes* A511 phage endolysin or Ply21 encoded by *Bacillus cereus *phage TP21, are also known [170-172]. A variety in the phage world allowed to develop endolysin-independent mechanisms of cell lysis, as well. Small ssDNA phage X174 and ssRNA phage Qβ encode special protein, called “protein antibiotics”, blocking host enzymes responsible for peptidoglycan biosynthesis thus releasing progeny without muralytic enzymes [34, 164, 173]. Despite all these cell destruction strategies, the most common way is still the first listed above strategy that forces bacteria to synthesize phage encoded lytic enzymes. Endolysins belong to a wider functional and structural group: bacterial cell wall hydrolases consisting of enzymes that degrade peptidoglycan encoded by different organisms: animals (named lysozymes), bacteria (autolysins) and phages (endolysins) [174]. Endolysins (also called lysins or muralytic hydrolases) are highly evolutionarily advanced enzymes encoded by large double-stranded DNA phages. Lysins have been characterized by no toxicity, different activity spectrum, low probability of resistance development and high efficiency. In contrast to typical antibiotics, phage endolysins are extremely effective biological compounds killing bacteria in seconds [20, 174-176]. Moreover, phage lysins can be utilized as biotechnological tools for selective bacterial detection or food preservation [174]. 

Phage lysins can be classified according to their structure as globular (built of a single, catalytic domain) or modular composed of two domains: (i) N-terminal catalytic domain (CD); and (ii) C-terminal cell wall binding domain (CWBD) [174, 177-179], or three domains (with an additional mid protein domain with enzymatic activities) [180]. Generally, endolysins from Gram-negative specific phages are characterized by a globular, single module structure whereas a additional substrate-binding domain is typical of Gram-positive specific phages [174, 181]. There are also some exceptions, like* P. aeruginosa* bacteriophages KZ144 and EL188 endolysins and OBPgp279 (from *P. fluorescens* phage OBP), 201φ2-1gp229 (encoded by *P. chlororaphis* phage 201φ2-1) or PVP-SE1gp146 (*S. enterica* phage PVP-SE1) exhibiting modular structure with an inverse arrangement of domains, N-terminal cell wall binding domain and a C-terminal CD [182]. The characteristics of selected endolysins are presented in Table **3**.

**Fig. (2):**
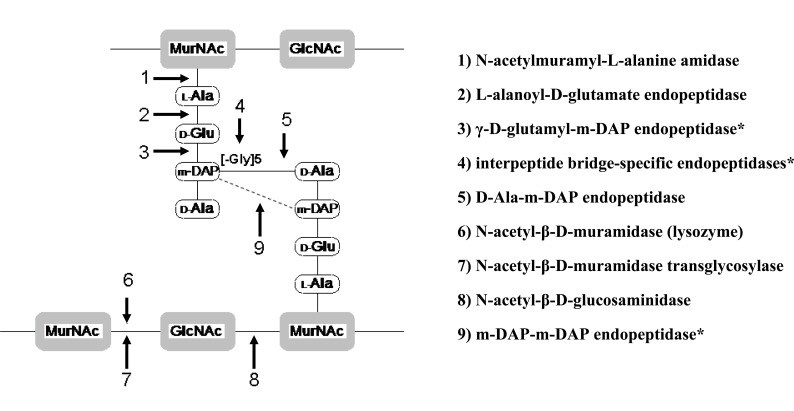
Known or proposed* mode of action of phage endolysins. GlcNAc - N-acetyl glucosamine; MurNAc - N-acetyl muramic acid; m- DAP - meso-diaminopimelic acid; L-Ala - L-alnine; D-Glu-D-glucosamine; [-Gly]_5_ - five glycine *residues* (characteristic for *S. aureus*).

Depending on the murein substrate properties (type of targeted bond in peptidoglycan) CD can exhibit different enzymatic activities. There are at least nine positions within murein where phage endolysins are known or are proposed to cleave (Fig. **2**) [179, 183, 184]. Most of the known and described phage encodes endolysins possess one type of enzymatic activities but some of them, especially large-phage endolysins, can exhibit more than one mode of action. Two independent CD enzymatic activities have been reported for LysK endolysin encoded by φ11 *S. aureus* specific phage and several endolysins derived from other staphylococcal bacteriophages: φMR11, φWMY, B30, NCTC 11261 and mycobacteriophage Ms6 [176, 183, 185, 186]. CWBD of bacteriophage-encoded endolysins is responsible for rapid-kinetic binding to specific carbohydrate epitope of peptydoglycan. CWBDs are characterized by high species specificity through the interaction only with the particular serovar group designations within a single species (CWBD of Ply118 endolysins binds to *L.* *monocytogenes* serovars 1/2, 3, and 7 or CWBD of Ply500 endolysin recognize only cells of serovars 4, 5, and 6 [187]. Because of these unique properties, phage binding domains can be widely used in biotechnology for bacteria detection [174, 182], immobilization and separation of bacterial cells [188]. They also have great potential in creating new classes of drugs targeted at a certain pathogen. 

The ability of purified endolysin as recombinant proteins to kill bacteria was first reported in 1959 [189]. Since then, research on phage endolysins, in particular encoded by Gram-positive bacteriophages has highly evolved. Strong and specific *in vitro* bactericidal efficiency has been described for many lysins like: PlyC from C1 bacteriophage, whose enzyme was found to kill *in vitro* each of the 10 tested Group A streptococcal strains with high efficiency [190] or Pal amidase derived from Dpl-1 phage, with strong lysis effect for 15 distinct clinical strains of *S. pneumoniae* [191]. *Another endolysin belonging to *Cp-1 phage was proved to be an efficient antibacterial agent causing rapid death for 19 tested pneumococcal strains, including three highly penicillin-resistant clones [192]. Antibacterial properties of Gram-negative phage endolysins have also been confirmed. Modular endolysins KZ144, EL188 and OBPgp279 driven from *Pseudomonas *phages were able to overcome the outer membrane barrier and kill *P. aeruginosa* strains including multi-drug resistant isolates [178, 182]. *Endolysins *exhibiting *a broad spectrum are generally* amidases encoded by Gram-negative specific phages because of very common presence of the amide bond between N-acetylmuramic acid and L-alanine in Gram-negative bacterial murein [176]. Recently, several globular Gram-negative phage endolysins with broad spectrum activity have been described. In combination with EDTA, all of these enzymes could reduce the number of *P. aeruginosa* PAO1, *S.* Typhimurium and *E. coli* XL1-Blue K [181]. A broad spectrum of activity may also be characteristic of some Gram-positive phages which encode amidase and non-amidase murein hydrolases, such as PlyV12 amidase of *Enterococcus faecalis* phage φ1 showing an antibacterial spectrum against *E. faecalis*, *E. faecium,* *S. pyogenes*, Groups B and C streptococci and *S. aureus *strains [193]*.* Mur-LH muramidase of *Lactobacillus helveticus* phage φ-0303 exhibits lytic activity against 10 different bacterial species [194].

It is assumed that the occurrence of bacterial resistance to phage lysins is unlikely. This theory has been evidenced for PlyG lysin and Pal amidase where no recovery of resistant strains was noticed even after 40 and 10 culture cycles respectively [191, 195]. *In vivo* therapy of murine bacterial infection with phage endolysin application was reported in 2001 by Nelson and co-workers [190]. Since then numerous publications have showed evidence of endolysins efficacy *in vivo *[190-192, 195-200]. In animal experiments, endolysins exhibited therapeutic activity regardless of the route of administration (intraperitoneally, intravenously, subcutaneous injections or orally). In contrast to not immunogenic antibiotics, phage endolysins applied intravenously stimulate a fast immune response and exhibit short half-life (15-20 min), however generated intibodies were not efficient in endolysin inactivation [20, 191, 192]. To avoid rapid inactivation of phage endolysin by antibodies, endolysins could be delivered frequently, or conjugated to polyethylene glycol reducing antibody binding [20]. More details concerning bacteriophage endolysins can be found in comprehensive reviews published in recent years [19, 20, 174, 176, 183, 184, 191].

### 4.4 Holins - Phage Enzymes Disturbing Cell Membrane 

Bacteriophage holins were defined two decades ago [201] and there is still little known about the structure, mechanism of action of these proteins and the structure of the holin-dependent lesions. Holins are small hydrophobic proteins with one to three transmembrane domains (due to the number of transmembrane holins are put into three classes). Class I holins have three transmembrane domains (TMD) with the N-terminus in the periplasm and the C-terminus located towards the cytoplasm. Phage λ holin is an example of a class I holin. Class II holins (holin gene of lambdoid phage 21) comprise two TMDs in an N-terminus and C-terminus in the periplasm. A class III (for example phage T4 holin) are holins with one TMD and an exceptionally large periplasmic domain [202, 203]. During the lytic cycle, holins accumulate in the cytoplasmic membrane of the host and their task is to disrupt it in an optimal, genetically determined time in the terminal stage of the lytic cycle, allowing the phage endolysin to escape and degrade peptydoglycan [204]. It can be said that holins determine the length of the infection cycle of ds DNA bacteriophages. Holins have even been called the smallest and simplest biological timers [46]. Here is the end of common features of phage holins because they are considered to be the most diverse functional group in nature [205]. Nevertheless, next to endolysin, holins are mentioned as the second essential programmed lysis protein. In the majority of phage genomes endolysin and holin genes are often adjacent with other genes: Rz and Rz1 encoded auxiliary enzymatic proteins, creating so called “lysis cassette”. The antibacterial potential of holins has not been yet studied in detail because of a lack of the experimental model for molecular expression of these highly toxic proteins [202], however, due to their capability of rapid perforation of biological membrane, these enzymes require special attention.

## 5 MANUFACTURING 

Large scale production is still an elaboration nevertheless the major principles of phage formula preparation have been described and discussed by recently published reviews [48, 52, 57]. The list of selected companies currently involved in phage preparation manufacturing are listed in Table **4**. In comparison to phage manufacturing some of the biggest pharmaceutical companies have ceased their discovery efforts of new antimicrobials because of a high probability of fast development in bacterial resistance and a high cost of clinical trials. Notwithstanding this, there is still intensive research on novel antibiotics conducted by Novartis, AstraZeneca, Merck, Pfizer, and Johnson & Johnson [22].

## CONCLUSIONS

Widespread emergence of resistance among common pathogenic bacteria has forced the scientific community to focus and develop alternatives to conventional antibiotic therapy. Phages have been proposed as natural antimicrobial agents to fight bacterial infections in humans, in animals or in crops of agricultural importance. The intensive research on phage biology has led to increasing potential phage application in different aspects of human activity. Possible applications of bacterial viruses and phage-encoded may concern: (i) phage therapy proteins; (ii) phage typing; (iii) bacterial detection; (iv) disinfection of medical tools and devices; (v) food decontamination; and (vi) drug delivery (vehicles). 

Major features of antibiotics, lytic bacteriophages and phage proteins as antimicrobials are summarized in Table **5.** The utilization of phage or phage proteins has both advantages and limitations. Phages are extremely specific, which requires detailed diagnostics of etiological factors causing infection. On the other hand, the narrow specificity protects the endogenous microbial flora. Therapeutic phages need to be precisely characterized in terms of biology and genetic features, because the use of not well defined lytic phages may lead to the expression of undesirable virulence factors as an adaptive response to phage infection and must be carefully studied prior to any clinical trials. A possible alternative to environmentally isolated phage utilization is the idea of genetically engineered phages construction exhibiting desirable properties without unexpected and potentially dangerous features. It is noteworthy that the use of only modified phages would also help to reduce the use of antibiotics. It seems that the application of phage-encoded proteins instead of environmentally isolated phages is more promising in terms of a broader activity spectrum, better tissue penetration, lower immunogenicity and low probability of bacterial resistance. Due to all these properties, lysins, for example, are effective in specific bacteria diagnostics and detection, phytopathogens elimination and in food preservation. Phage lysins can also support or replace antibiotics in the future, especially since these enzymes have been used, with successful results, to treat Gram-positive bacterial infections in animal models. Previous studies have confirmed the effectiveness of phage lysins *in vitro* and *in vivo *using animal models. The other phage proteins, as highly effective inhibitors of bacterial replication, transcription or protein synthesis, may be applied in the future after successful delivery into bacterial cytoplasm. This could be achieved by genetically engineered phages contribution carrying early protein encoding genes or by application of other vehicles such as targeted fluid liposome encapsulated proteins fusing with bacterial envelopes. Manufacturing phage proteins is relatively simple by exploitation of molecular engineering allowing production of recombinant peptides or synthetic analogues. Large scale production and purification methods are well developed in other pharmaceutical branches working on proteins application. The most important aspect of phage or phage proteins application in therapy is the possible combination of multi-agent treatment including antibiotics, phages and phage proteins. These inventions of biological-chemical treatment may minimize the chance of developing residence and reduce the incidence of phage resistance and/or antibiotic resistance. 

## Figures and Tables

**Table 1.** Examples of Phage-Associated Polysaccharide Depolymerases.

Activity	Protein	Origin (phage)	Host	Reference(s)

**Endosialidases**	endoNA	φK1A	*E. coli* K1	[95]
	endoNE	φK1E	*E. coli* K1	[96, 97]
	endoNF	φK1F	*E. coli* K1	[98, 99]
	endoN63D	φ63D	*E. coli* K1	[100]
	endoNK1-5	φK1-5	*E. coli* K1	[93]
	endoNK1	CUS-3	*E. coli* K1	[101]

**Hyaluronian lyases**	hylP1	H4489A	*S. pyogenes* SF370	[112]
	hylP2	10403	*S. pyogenes* SF370	[113, 114]
	hylP3	ND^*a*^	*S. pyogenes* serotype M1	[115, 116]
	SEQ2045	ND	*S. equi* 4047	[117]

**Other lyases**	Kfl5	φK5A	*E. coli* K5	[206]
	K5 lyase	φK1-5	*E. coli* K5	[93]
	ElmA	prophage	*E. coli* K5	[207]
	alginate lyase	φ15	*P. putida*	[81]
	alginate lyase	2	*P. aeruginosa*	[122]
	lyase	no name	*.vinelandii*	[208]
	lyase	JA1	*V.cholerae* O139	[78]

**Table 2.** Phage Early Proteins Target Various Essential Mechanisms in the Host Bacterial Cell.

Host mechanism	Phage	Host	Phage protein	Host protein	Stage	Effect	Reference(s)
**Replication**	λ	*E.coli*	CIII	FtsH	Early	Competitive inhibition of FtsH	[209]
			Gam	RecBCD	delayed early	Inhibition of nuclease and helicase activities	[210]
	77	*S. aureus*	gp104	DnaI (helicase loader)	Early	Inhibition of host replication	[147]
	Twort	*S.aureus*	gp168	DnaN	ND	Shutoff of host replication	[147]
	G1	*S.aureus*	gp240	DnaN	ND	Shutoff of host replication	[147]
	N4	*E.coli*	gp8	HolA (DNApol III δ subunit)	ND	Shutoff of host replication	[211]
**Transcription**	φEco32	*E.coli *55	gp36 (α factor)	RNAP complex	ND	Rocognition of phage promoters	[153]
	φEco32	*E.coli *55	gp79	RNAP complex	Early	Inhibition of host transcription	[153]
	T7	*E.coli*	gp0.7 C-term	RNAP β’ subunit	Early	Host transcription shutoff	[212]
			gp0.7 N-term	RNAP β and β’ subunits	ND	Efficiency of termination	[213]
			gp0.7 N-term	RNAse III	ND	Processing of T7 mRNA	[214]
			gp0.7 N-term	RNAse E and RhlB	ND	Protection of T7 mRNA from degradation	[215]
			gp2	RNAP β’ subunit	ND	Inhibition of transcription initiation	[216]
	T4	*E.coli*	Alc	RNAP β subunit	Early	Host transcription shutoff	[217]
			ModA	Both RNAP β subunits	Early	Lower expression of T4 early and host genes	[218]
			AsiA	σ^70^ factor	Early	Inhibition of σ^70^ promoter recognition	[219]
			MotA	σ^70^ factor	Early	Recognition of T4 middle promoters	[219]
			RpbA	RNAP core enzyme	Early/middle	Stimulation of T4 late genes expression	[220]
			Mrh	σ^32^	Early	Decoy of σ^32 ^from RNAP core	[221]
			Srh	RNAP core enzyme	Early	Decoy of σ^32 ^from RNAP core	[221]
			Srd	RNAP core enzyme	Early	Decoy of σ^70^/σ^38^ from RNAP core	[221]
	λ	*E.coli*	N	RNAP core, NusA,NusG	Early	Antitermination, delayed early transcription	[222]
			Q	RNAP holoenzyme	delayed early	Antitermination, late transcription	[223]
	P2	*E.coli*	Org	RNAP α subunit	Early	Inhibition of late transcription	[224]
	SPO1	*B. subtilis*	gp44	RNAP β subunit	Early	Inhibition of host RNAP	[225]
			gp28	RNAP core enzyme	Early	σ factor for middle transcription	[226]
	Xp10	*E.coli*	P7	RNAP β’ subunit	Early	Inhibition and antitermination of transcription	[227]
	P23-45	*T.thermophilus*	gp39	RNAP holoenzyme	ND	Shutoff of host transcription	[227]
			gp76	RNAP holoenzyme	ND	Shutoff of host transcription	[227]
**Translation**	T4	*E.coli*	ModB	30S ribosomal subunit S1,TF	Early	Promotes binding of mRNAs	[158, 228]
			Alt	EF-Tu, TF	ND	Aminoacyl-tRNAs recruitment	[158, 228]
	T7	*E.coli*	gp0.7	IF1,IF2,IF3	ND	Helper function for IF2 and IF3	[228]
	φYS40	*T.thermophilus*	ND^a^	IF2,IF3	Early	Translation of phages leaderless mRNAs	[155, 156]
**Cell division**	Rac	*E.coli*	Kil	FtsZ	Early	Cell division arrest	[161]

a ND: non determined

**Table 3.** The Characteristics of Selected Endolysins.

Bacteria	Phage	Host	Phage protein	Name (displayed activity)	Reference(s)
	C_1_ phage	*S. pyogenes*	modular	PlyC (amidase) *	[177, 190]
	Dp-1 phage	* S. pneumoniae*	modular	Pal (amidase) *	[191, 229]
** Gram-positive**	Cp-1 phage	*S. pneumoniae*	modular	Cpl-1 (muramidase) *	[192, 230, 231]
	γ phage	*B. anthracis*	modular	PlyG (amidase)	[195, 232]
	φBcp1 phage	*B. anthracis*	modular	PlyB (muramidase) *	[233]
	B4 phage	*B. cereus*	modular	LysB4 (L-alanoyl-D-glutamate endopeptidases) *	[234]
	BPS13 phage	*B. cereus*	modular	LysBPS13 (amidase)	[235]
	φA118 phage	*L. monocytogenes*	modular	Ply118 (L-alanoyl-D-glutamate peptidase)	[170, 187, 236]
	φA511 phage	*L. monocytogenes*	modular	Ply511 (amidase)	[170, 236]
	φA500 phage	*L. monocytogenes*	modular	Ply500 (L-alanoyl-D-glutamate peptidass)	[170, 187]
	NCTC 11261 phage	S. agalactiae	modular	PlyGBS (endopeptidase, muramidase)	[196]
	φB30 phage	*S. agalactiae*	modular	GBSlysine (endopeptidase, glycosidase)	[237, 238]
	φP68 phage	*S. aureus*	modular	Lys16 (endopeptidase, amidase)	[239]
	Phi Twort	*S. aureus*	modular	PlyTW (amidase)*	[240]
	φ11 phage	*S. aureus*	modular	Lysϑ11 (amidase, peptidase)*	[241- 243]
	φMR11 phage	*S. aureus*	modular	MV-L (amidase)	[198]
	φH5 phage	*S. aureus*	modular	LysH5 (amidase, peptidase)	[244]
	φK phage	* S.aureus*	modular	LysK (amidase, endopeptidase) *	[180, 245, 246]
	GH15 phage	*S. aureus*	modular	LysGH15 (amidase)	[200]
	SAP-1 phage	* S.aureus*	modular	SAL-1 (amidase, peptidase)	[246]
	P-27/HP phage	*S. aureus*	modular	P-27/HP lysin	[247]
	vB_SauS-φIPLA88	*S. aureus*	modular	HydH5 (lysozyme, peptidase)	[248]
	2638A phage	*S. aureus*	modular	2638A endolysin (peptidase, amidase)	[249]
	φSMP phage	*S. suis*	modular	LySMP (endopeptidase, glycosidase)	[250]
	φWMY M phage	*S. warneri*	modular	LysWMY (amidase, peptidase) *	[251]
	φ1 phage	E. faecalis	modular	PlyV12 (amidase)	[193]
	EFAP-1 phage	*E. faecalis*	modular	EFAL-1 (amidase)	[252]
	φEF24C phage	*E. faecalis*	modular	ORF9 (amidase)	[253]
	F168/08 phage	*E. faecalis*	modular	Lys168 (peptidase)	[254]
	F170/08 phage	*E. faecalis*	modular	Lys170 (amidase)	[254]
	φ-0303 phage	*L. helveticus*	modular	Mur-LH (muramidase) *	[194]
	mu1/6.	*S. aureofaciens*	modular	mu1/6Lyt (amidase)	[255]
	CMP1 phage	*C. michiganensis*	modular	LysCMP1peptidase	[256]
	CN77 phage	*C. michiganensis*	modular	LysCN77peptidase	[256]
	φ3626 phage	*C. perfringens*	modular	Ply3626 (amidase)	[257]
	φCD27 phage	*C. perfringens*	modular	CD27L (amidase) *	[258]
	φCP39O phage	*C. perfringens*	modular	PlyCP39O (amidase)	[259]
	φCP26F phage	*C. perfringens*	modular	PlyCP26F (amidase)	[259]
	φCTP1 phage	*C. tyrobutyricum*	modular	CTP1gp29 (amidase)	[260]
	φSM101 phage	*C. perfringens*	modular	Psm (muramidase)	[261]
** Gram-negative**	φAB2 phage	*A. baumannii*	modular	LysAB2	[262]
	201φ2-1 phage	*P. chlororaphis*	modular	201ϑ2-1gp229 (goose-like lysozyme)	[182]
	φKZ phage	*P. aeruginosa*	modular	KZ144 (lytic transglycosylase and lysozyme)	[166, 178]
	EL phage	*P. aeruginosa*	modular	EL188 (lytic transglycosylase)	[166]
	PaP1	*P. aeruginosa*	modular	PaP1_gp072 (unknown enzymatic function)	[263]
	φKMV	*P. aeruginosa*	modular	KMV36C (muramidase)	[264]
	OBP phage	*P. fluorescens*	modular	OBPgp279 (lysozyme)	[182]
	PVP-SE1 phage	*S. enterica*	modular	PVP-SE1gp146 (lysozyme)	[182]
	SPN1S phage	*S. *Typhimurium	modular	SPN1S endolysin (lysozyme)	[265]
	BcepC6B phage	*B. cepacia*	globular	BcepC6gp22 (lytic transglycosylase)	[181]
	T5 phage	*E. coli*	globular	LysT5 (peptidase)*	[266]
	P2 phage	*E. coli*	globular	P2gp09 (lytic transglycosylase)	[181]
	PsP3 phage	*S. enterica*	globular	PsP3gp10 (lytic transglycosylase)	[181]
	K11 phage	*K. pneumoniae*	globular	K11gp3.5 (amidase)	[181]
	KP32 phage	*K. pneumoniae*	globular	KP32gp15 (amidase)	[181]

* endolysins biochemically characterized for the actual peptidoglycan cut site.

**Table 4.** Selected Commercial Companies Involved in the Development of Phage-Based Products.

Bacteriophages or phage proteins	Product(s) Stage of development	Application	Company/ Web site address	Location
**Phage**	Phage tablet (Phagedys, Phagetyph, Phagesal); Liquid (Phagesti, Phagyo, Phagestaph, Phagepy)	Treatment and prophylaxis of gastrointestinal infections (*Shigella, Salmonella, E. coli, Proteus, Staphylococcus, Pseudomonas, Enterococcus* and their combinations) Treatment and prophylaxis of purulent inflammatory infections (*Streptococcus, Staphylococcus, Pseudomonas, Proteus* and their combinations)	JSC Biochimpharm http://www.biochimpharm.ge	Tbilisi, Georgia
**Phage**	Phage tablet (Intestiphage) and liquid (Pyophage), patented and licensed	Treatment and prophylaxis of gastrointestinal infections (*Shigella, Salmonella, E. coli, Proteus, Staphylococcus, Pseudomonas, Enterococcus* ) Treatment and prophylaxis of purulent inflammatory infections (*Streptococcus, Staphylococcus, Proteus*)	Biopharm-L http://www.biopharm.ge that owns Advanced Biophage Technologies http://advancedbiophagetechnologies.com	Tbilisi, Georgia
**Phage**	PhagoBioDerm® - biodegrable polymer film	Treatment of wounds, bedsores, tropic ulcers and different origin burns	PolymerPharm http://www.polymerpharm.ge	Tbilisi, Georgia
**Phage**	BioPhage-PA (phase II trial completed), BioPhage-PR (pre-clinical)	Treatment of *P. aeruginosa* in chronic infections of the ear and lung associated with CF	AmpliPhi Biosences Corporatian http://www.ampliphibio.com	Washington, USA
**Phage**	Research and development	Environmental therapies and diagnostics, products geared towards antibacterial resistance problems and as a weapon against bioterrorism	Biophage Pharma Inc., http://biophagepharma.net	Montreal, Canada
**Phage**	LISTEX P100^TM^, product available; SALMONELEX^TM^, temporary use exemption	Prevention of the outgrowth of *Listeria* strains on food during processing	Micreos Food Safety http://www.micreosfoodsafety.com	Wageningen, Netherlands
**Unknown**	unknown	Phage solutions for environmental, cosmetics and medical bacterial infections	Innophage, Ltd http://innophage.com	Porto, Portugal
**Phage**	ListShield^TM^(LMP-102), EcoShield^TM^ (products available); PLSV-1^TM^, INT-401^TM^ against *Salmonella* and *Clostridium* in poultry, respectively (licensed out); SalmoShield^TM^, ShigaActive^TM^ (in development)	Eliminating or reducing contamination of food with *L. monocytogenes* and *E. coli* O157:H7, respectively	Intralytix http://www.intralytix.com	Baltimore, USA
**Phage**	Phage gel (pre-clinical)	Prevention and treatment of infections caused by methicilin-resistant strains of *S. aureus* (MRSA) and *C. difficile*	Novolytics http://www.novolytics.co.uk	Coventry, UK
**Phage**	AGRIPHAGE (product available) research and development	Treatment of harmful bacteria (*Xanthomonas sp., Pseudomonas sp*.) on tomato and pepper plants; Protection of food and water resources (*E.coli, Listeria, Salmonella*)	OmnyLitics http://www.omnylitics.com	Salt Lake City, USA
**Unknown**	SASP ject^TM^	Delivering of lethal proteins	Phico Therapeutics, Ltd. http://www.phicotherapeutics.co.uk	Cambridge, UK
**Phage**	Prototype products, some entering clinical trials	Treatment of MRSA, VRE (vancomycin-resistant enterococci) and multi-resistant *P. aeruginosa *infections	Special Phage Holdings Pty Ltd http://www.specialphageservices.com.au	Brookvale, Australia
**Phage (modified)**	Viridax^TM^ (pre-clinical)	Treatment of *S. aureus* and other staphylococcal infections	Viridax^TM^ http://www.viridax.com	Baco Raton, USA
**Phage**	Staphage lysate (SPL®)	Treatment of canine pyoderma and related staphylococcal hypersensitivity, or staphylococcal skin infections	Delmont Laboratories, Inc. http://www.delmont.com	Swarthmore, USA
**Phage**	Stafal	Topical application in infections caused by staphylococcal strains	Sevapharma http://www.sevapharma.cz	Praha, Czech Republic
**Phage**	Bacteriophagum, Piobacteriophagum	Treatment of various bacterial infections	Immunopreparat Research Productive Association	Ufa, Russian Feredation
**Recombinant proteins**	Artilysins^TM^	Clinical applications	Lisando GmbH http://www.lisando.com	Regensburg, Germany
**Recombinant proteins**	StaphTAME also known as P128 (pre-clinical completed)	Prevention and treatment of staphylococcal infections, including MRSA	GangaGen http://www.gangagen.com	Bangalore, India; Palo Alto, USA; Canada
**Recombinant proteins**	hyaluronate lyases (hylp1, hylp2, hylp3)	Use in carbohydrate research	Prozomix http://www.prozomix.com	Northumberland, UK
**Phage**	SmartPhage^TM^	Treatment of MRSA and *P. aeruginosa* infections	Special Phage Service Pty Ltd http://www.specialphageservices.com.au	Brookvale, Australia
**Phage**	FASTPlaque-response^TM ^FASTPlaqueTB ^TM^	Detection of rifampicin resistance in smear-positive sputum specimens containing *M. tuberculosis*, for detecting of *M. tuberculosis* in human sputum samples	Biotec Laboratories Ltd. http://www.biotec.com	Suffolk, UK
**Phage**	MicroPhage MRSA/MSSA test	Differentiation of MRSA and MSSA (methicilin-susceptible *S. aureus*)	Microphage, Inc. http://www.micro-phage.com	Longmont, USA
**Phage**	*E. coli* K1 and K5 phage suspensions	Differentiation of K1 and K5 capsule polysaccharides antigens	Statens Serum Institut http://www.ssi.dk	Copenhagen, Denmark
**Unknown**	Unknown	Indicator of the presence of rare taxa in a selected test substrate and for determining the relatedness of the isolated taxa	Micropeace	Melbourne, Australia

**Table 5.** Major Features of Antibiotics, Lytic Bacteriophages and Non-Lytic Phage Proteins as Antimicrobials.

Selected feature	Antibiotic	Phage	Non-lytic phage protein
** Selective toxicity**	Active only on specific microbial metabolism pathway; Inhibition of bacterial toxins production possible	Propagation on bacterial host (predator-prey relation)	Active only on specific microbial metabolism pathway; Inhibition of bacterial toxins production possible
** Antimicrobial spectrum**	Broad against Gram- positive and Gram-negative, extra- and intracellular pathogens	Narrow very specific mostly on one bacterial species; No available phage preparations for intracellular pathogens	Narrow or broad
** Influence on normal flora**	Dysbiosis; Secondary infection possible	No influence eradication of targeted strain only	Low influence on gut flora
** Serious side effects on the host**	Allergy, dysbiosis, secondary infections; Endotoxin (LPS) and other toxins release possible	Endotoxin (LPS) and other toxins release during cell lysis possible	Endotoxin (LPS) and other toxins release during cell lysis possible
** Efficient bacterial killing**	Bacteriostatic or bactericidal; Concentration- or time-dependent killing; PAE effect possible (postantibiotic effect); MIC (minimum inhibitory concentration); Effective on growing cells	Bacteriolytic; Phage titer-dependent killing; Virulence efficacy: MOI, burst size, growth rate; Effective on growing cells	Bacteriostatic or bacteriolytic; Concentration- dependent killing; MIC (minimum inhibitory concentration); Effective on growing and non-growing cells
** Penetration to the tissues, concentration, dose, timing of administration**	Well defined; Blood flow to tissue; Chemical structure affects penetration and plasma protein binding - effective concentration; Relatively long shelf-life; Concentration at the infection site related on systemic concentration and blood circulation	Not well defined; Size and capsid protein structure affects systemic concentration regulated by reticuloendothelial system (RES) clearance and immune cellular defense mechanisms; Self-replicating agent - the concentration increase at the infection site	Not well defined; Blood flow to tissue; Chemical structure affects penetration, plasma protein binding and proteolysis degradation - effective concentration; Concentration at the infection site related on systemic concentration and blood circulation
**Stability**	Well elaborated; Chemically-stable	Not much data in current papers	Relatively not stable;
** Formulations**	Easy to administrate - pills, syrups, injections, aerosols, formulas for local application	Liquid phage filtrate, tablets, formulas for local application	Injections, aerosols, formulas for local application
** Delivery route**	Orally or parenteral route (mostly intravenous) for majority of infection locations (systemic or topical disease); Locally (topical infections)	Parenteral route (systemic infections); Orally (gastrointestinal infections); Locally (topical infections)	Parenteral route (systemic infections); Oral application limited by proteolysis; Locally (topical infections)
**Resistance development**	Vertical - mutation and selection; Horizontal - acquisition of resistance genes from another organism via transformation, transduction and conjugation Multidrug resistance acquisition possible; High level of induced resistance	Vertical - mutation and selection; Temperate phage acquisition; Low level of induced resistance	Vertical - mutation and selection; Low level of induced resistance
**Multidrug therapy**	drugs in combined therapy; Prevention of resistance development; Eradication of multidrug resistant strains; Synergistic effect possible	Cocktail of phages (3-5) or phage-antibiotic combination; Prevention of resistance development; Extended activity spectrum; Synergistic effect possible	Combined therapy of protein-protein; phage-protein; antibiotic-protein; antibiotic-phage-protein; Prevention of resistance development; Extended activity spectrum; Synergistic effect possible
**Development of new preparation**	Antibiotic modification; *In silico* design possible	Fast and easy isolation of new phages from environmental source	*In silico* development by protein data bases exploration; Analysis of annotated phage genomes
**Biofilm eradication**	Difficult effective drug concentration in biofilm structure limited;	Relatively effective phage penetration into the biofilm structure possible by means of EPS degradation (phage enzymes)	Effective biofilm degradation possible by EPS degrading phage enzymes
**Manufacturing**	Well elaborated	Limitation in densification and purification; Large scale methods need to be adopted	Relatively simple; Recombinant peptides or synthetic analogues; Large scale methods adopted
